# Identifying Patients with Nonalcoholic Fatty Liver Disease in Primary Care: How and for What Benefit?

**DOI:** 10.3390/jcm12124001

**Published:** 2023-06-12

**Authors:** Andrew D. Schreiner, Naveed Sattar

**Affiliations:** 1 Department of Medicine, Medical University of South Carolina, 171 Ashley Ave, Charleston, SC 29425, USA; 2 Institute of Cardiovascular & Medical Sciences, University of Glasgow, 126 University Place, Glasgow G12 8TA, UK

**Keywords:** nonalcoholic fatty liver disease, primary care physicians, primary health care, weight loss

## Abstract

Despite its increasing prevalence, nonalcoholic fatty liver disease (NAFLD) remains under-diagnosed in primary care. Timely diagnosis is critical, as NAFLD can progress to nonalcoholic steatohepatitis, fibrosis, cirrhosis, hepatocellular carcinoma, and death; furthermore, NAFLD is also a risk factor linked to cardiometabolic outcomes. Identifying patients with NAFLD, and particularly those at risk of advanced fibrosis, is important so that healthcare practitioners can optimize care delivery in an effort to prevent disease progression. This review debates the practical issues that primary care physicians encounter when managing NAFLD, using a patient case study to illustrate the challenges and decisions that physicians face. It explores the pros and cons of different diagnostic strategies and tools that physicians can adopt in primary care settings, depending on how NAFLD presents and progresses. We discuss the importance of prescribing lifestyle changes to achieve weight loss and mitigate disease progression. A diagnostic and management flow chart is provided, showing the key points of assessment for primary care physicians. The advantages and disadvantages of advanced fibrosis risk assessments in primary care settings and the factors that influence patient referral to a hepatologist are also reviewed.

## 1. Introduction

Nonalcoholic fatty liver disease (NAFLD) is a leading cause of chronic liver disease worldwide [1], with an estimated global prevalence of around 32.4% [2]; however, further studies are needed to provide more accurate prevalence data. While regional prevalence estimates may vary [1,2,3,4], NAFLD is a major contributor to liver-related morbidity and mortality worldwide [5]. Alongside a rise in prevalence, NAFLD has emerged as a driver of cirrhosis and hepatocellular carcinoma (HCC) and is increasing the need for liver transplantation [6,7,8]. NAFLD is characterized by the buildup of fat in the liver, or “steatosis”, in the absence of other causes of secondary hepatic steatosis, such as alcohol consumption, viral hepatitis, or other chronic liver diseases [9]. In this way, NAFLD is a form of ectopic fat, often linked to excess fat in other ectopic tissues such as the blood vessels, the heart, and the pancreas [10], although steatosis alone does not define liver disease. Excess ectopic fat perturbs triglyceride synthesis and glucose metabolism and is linked to a higher vascular risk [10]. Consequently, NAFLD is associated with conditions of metabolic dysfunction, including type 2 diabetes (T2D), obesity, hypertension, and dyslipidemia [1,11]. The coexistence of these metabolic conditions with NAFLD, specifically T2D and obesity, is associated with severe liver-related outcomes, such as nonalcoholic steatohepatitis (NASH), advanced fibrosis, cirrhosis, and mortality [9,12,13,14,15,16,17].

Despite the prevalence and growing burdens of morbidity and mortality, NAFLD is under-diagnosed in real-world primary care settings [18,19,20]. The factors contributing to this under recognition likely include the absence of recommendations for systematic routine NAFLD screening, the uncertainties related to the currently available diagnostic tests, and the lack of pharmacologic therapies that are specifically approved for the reversal of NAFLD/NASH [9,21,22,23]. In addition, recommendations for NAFLD care have predominantly appeared in specialty journals [24], and many countries do not have primary care follow-up algorithms [25]. Thus, primary care physicians (PCPs) report unfamiliarity with, and limited access to, the currently available tools for assessing NAFLD disease progression and identifying patients at greatest risk of future poor health outcomes [26,27]. Due to the progressive nature of NAFLD, a timely diagnosis of the disease is considered important to provide healthcare practitioners the opportunity to motivate patients to make lifestyle changes that could mitigate disease progression and related conditions, such as diabetes. As the prevalence of NAFLD and related metabolic conditions is rising [28,29], patients with these conditions are becoming an increasingly larger cohort in primary care settings. Monitoring patients with known risk factors in primary care will, in turn, become increasingly important. PCPs, therefore, play a critical role in identifying and diagnosing patients with NAFLD, which will help to inform treatment management plans and may prevent disease progression.

This review will address the challenges that NAFLD presents and will reinforce the critical diagnosis and management strategies that physicians can perform in primary care settings. Specifically, we will emphasize the importance of diagnosing NAFLD, prescribing weight-loss interventions, addressing cardiovascular risk, and assessing for advanced fibrosis in patients diagnosed with NAFLD. A longitudinal, representative case study will illustrate the application of these management concepts in a primary care patient. The patient case described in this publication is fictional and does not represent actual events or a response from an actual patient. The authors developed this fictional case for educational purposes only.

## 2. Pathophysiology of NAFLD

The pathophysiology of NAFLD spans a continuum from simple steatosis with no, or minor, inflammation (nonalcoholic fatty liver (NAFL)) to steatosis, accompanied by inflammation (NASH), fibrosis, and cirrhosis [30]. However, Medicare claims data from the US indicate that, among patients initially diagnosed with NAFLD/NASH, there is a 39% probability of it progressing to more severe liver disease over an 8-year follow-up period [19]. It is worth noting that the relatively low incidence of NAFLD reported in the Medicare sample (5.7%) [19] may bias this estimated risk of disease progression. Steatosis is the key defining histologic feature across the NAFLD spectrum [30], and guidelines recommend evidence of steatosis for diagnosis (see review [31]). Metabolic dysfunction appears to be central to the pathological processes, including the progression of NAFL to NASH, fibrosis, cirrhosis, and HCC [10,30,32]. When there is an oversupply of calories and/or insufficient expenditure causing weight gain, an excess of fatty acids leads to the buildup of toxic lipids in the liver [10,33]. Such toxic lipids accumulate at different body mass index (BMI) thresholds, depending on individuals’ underlying comorbidities, genetics, ethnicity, sex, age, and body fat distribution [10]. Over time, these toxic lipids can trigger the inflammatory pathways that contribute to the development of steatosis, inflammation, and progressive liver damage [10]. Prognostically, advanced fibrosis is the most important histological feature that is looked for in patients with NAFLD, with liver-related morbidity and mortality increasing with each progressive fibrosis stage [34,35,36,37]. Thus, advanced fibrosis is a warning sign of serious liver disease.

In real-world settings, patients with NAFLD exhibit heterogeneity in the clinical presentation and disease course of their fatty liver disease [38]. Multiple factors, including age, biological sex, hormonal status, alcohol intake, smoking, and metabolic status, can contribute to NAFLD progression, with factors potentially working synergistically to contribute toward the disease course [38]. A recent review panel has suggested that the nomenclature of NAFLD may not be fully reflective of the complexities of factors influencing the metabolism and disease progression, suggesting that NAFLD be renamed as metabolic-associated fatty liver disease (MAFLD) [38], although this new abbreviation has not had a wide uptake.

**Figure 1:**
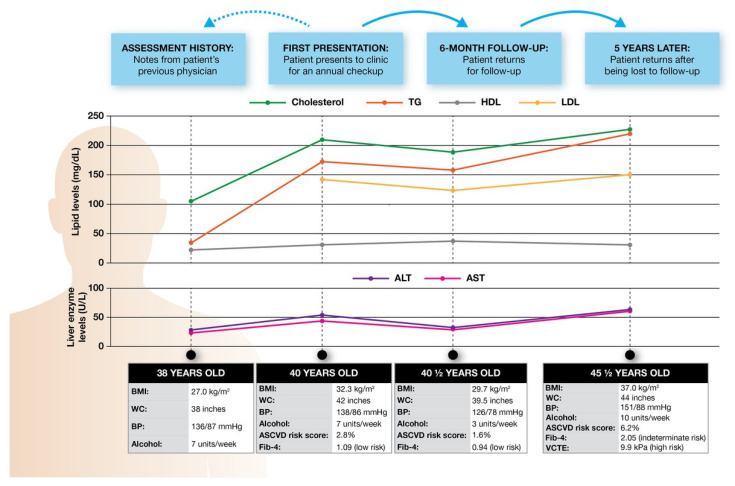
Longitudinal case study of a patient with NAFLD. ALT, alanine aminotransferase; ASCVD, atherosclerotic cardiovascular disease; AST, aspartate aminotransferase; BMI, body mass index; BP, blood pressure; Fib-4, Fibrosis-4; HbA1c, hemoglobin A1c; HDL, high-density lipoprotein; LDL, low-density lipoprotein; TG, triglyceride; VCTE, vibration-controlled transient elastography; WC, waist circumference.

**Case Study.** First presentation: A new male patient attends clinic for an annual checkup (Figure 1).

- Age: 40, BMI: 32.3 kg/m^2^, waist circumference: 42 inches, blood pressure: 138/86 mmHg, and consuming 7 units of alcohol per week;
- Cholesterol: 210 mg/dL, triglyceride: 174 mg/dL, high-density lipoprotein (HDL)-cholesterol: 31 mg/dL, and low-density lipoprotein (LDL)-cholesterol: 144 mg/dL;
- Alanine aminotransferase (ALT): 54 U/L, aspartate aminotransferase (AST): 44 U/L, and platelets: 220 K;
- Hemoglobin A1c (HbA1c): 6.1%;
- He is not taking any medications.

## 3. When to Pursue a NAFLD Diagnosis

### 3.1. Pursuing a NAFLD Diagnosis following Abnormalities in Aminotransferases or in Liver Imaging

**Figure 2:**
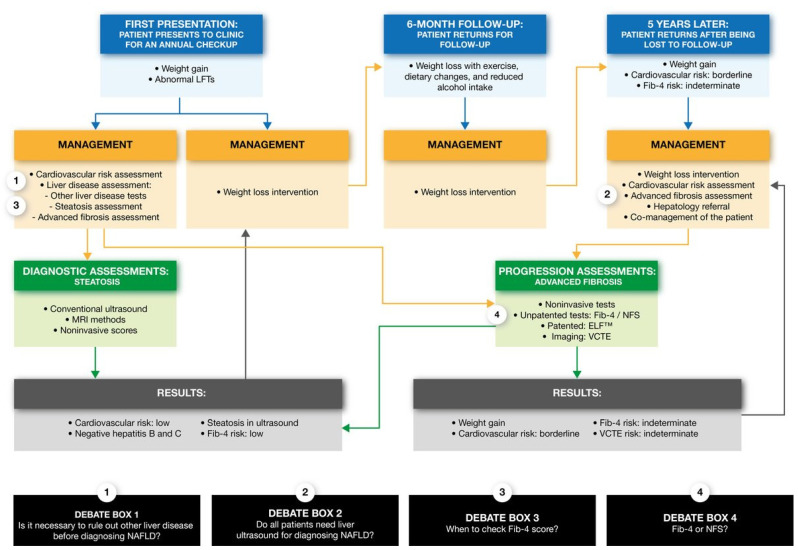
Diagnostic flow chart of patients with NAFLD in primary care. ELF™, enhanced liver fibrosis; Fib-4, Fibrosis-4; LFT, liver function test; NAFLD, nonalcoholic fatty liver disease; NFS, NAFLD fibrosis score; VCTE, vibration-controlled transient elastography.

NAFLD is generally suspected during routine clinical care when abnormalities are detected in serum aminotransferases (alanine aminotransferase (ALT) and, to a lesser extent, aspartate aminotransferase (AST)) or steatosis is identified through liver imaging (Figure 2) [18,39,40]. NAFLD is a common cause of incidentally detected aminotransferase abnormalities [41,42]. The British Society of Gastroenterology and the American College of Gastroenterology (ACG) guidelines for addressing ALT and AST abnormalities recommend excluding competing liver disease diagnoses by obtaining a thorough exposure (alcohol and drug), medication (including herbal supplements), and travel history [43,44]. Additionally, these specialty society guidelines recommend viral hepatitis testing (hepatitis B surface antigen and hepatitis C antibody), a serologic hemochromatosis assessment (ferritin and transferrin saturation), autoimmune hepatitis testing (anti-mitochondrial, anti-smooth-muscle, and anti-nuclear antibodies, as well as serum immunoglobulins), and imaging with an abdominal ultrasound (US) (Box 1) [43,44]. Furthermore, the ACG guidelines recommend serologic evaluations for Wilson’s disease and alpha-1 antitrypsin deficiency [44]; however, ceruloplasmin testing for Wilson’s disease may be unnecessary in patients >55 years of age, given the rarity of late-onset Wilson’s disease and the monetary costs involved in testing [45,46]. A more focused testing strategy that incorporates the pre-test probability of liver diseases has been studied and the results suggested that testing limited to assessing viral hepatitis, alcohol history, and US imaging can reduce the costs and limit the occurrence of false-positive test results [46].

Box 1 **Is it necessary to rule out other liver diseases before diagnosing NAFLD?**

   The current guidelines on abnormal liver function tests have adopted a “diagnosis of exclusion” strategy, recommending screening for and ruling out various causes of liver disease systematically. In primary care, the cost, waiting times, and availability may limit the utility of extensive screening strategies, particularly in patients at high risk for NAFLD. Taking a clinical history and recognizing risk factors is a practical first step to identifying at-risk patients and can help to determine how to proceed. In patients with abnormal serum ALT levels (or ALT levels near the high end of the normal range) and other features potentially consistent with NAFLD (such as excess adiposity or elevated triglyceride or HbA1c levels), one approach can be to recommend lifestyle changes without conducting further assessments. If the patients lose weight, and the ALT levels normalize or clinically meaningfully improve, a NAFLD diagnosis can be strongly suspected, especially if there are parallel improvements in related factors such as triglyceride and HbA1c levels [33]. If so, such findings would help to provide biochemical evidence of lower liver fat and alleviate the need for multiple expensive tests and the ensuing burden on the patient.

   An alternative approach in at-risk patients can be to limit testing to the most common causes of disease (i.e., performing a viral hepatitis assessment, an alcohol and medication history, and a liver US). NAFLD can then be diagnosed if the viral hepatitis assessments are negative, the alcohol history is not suggestive of alcohol-related liver disease, the medication history shows the patient is not on steatogenic medication, and steatosis is detected by the US.

Although elevated ALT and AST levels may be a useful signal for pursuing a NAFLD diagnosis, a portion of patients with NAFLD may also present with normal aminotransferase levels, along with other metabolic features. A recent systematic review estimated that 25% of patients with NAFLD present with ALT values within the “normal” range, mainly in females and patients with diabetes [47]. Therefore, while abnormalities in serum aminotransferases may indicate the presence of NAFLD, they should not be used as the sole diagnostic criterion.

Incidental identification of hepatic steatosis on abdominal imaging can also prompt the pursuit of a NAFLD diagnosis. However, several small studies suggest that, even when steatosis is noted on radiographic imaging reports in patients with metabolic risk factors, a formal diagnosis is infrequently made [20,48]. Once hepatic steatosis is identified, physicians can assess for metabolic risk factors and evaluate for secondary causes of hepatic steatosis, including alcohol consumption, viral hepatitis, and medications (e.g., tamoxifen, amiodarone, and corticosteroids) [9].

### 3.2. Pursuing a NAFLD Diagnosis in High-Risk Patients

The clinical practice guidelines for NAFLD recommend that PCPs should consider screening patients who are at high risk for NAFLD, specifically those with metabolic risk factors such as obesity and diabetes [21,22], or be aware of the higher risk of NAFLD in such patients [9,49], while diabetes guidelines recommend evaluating high-risk patients for NAFLD and fibrosis when they present with elevated ALT levels or hepatic steatosis on a US [50]. The key risk factors mentioned in the guidelines include T2D, obesity, dyslipidemia, hypertriglyceridemia, elevated ALT and gamma-glutamyl transferase, and male sex [31]. Recent machine learning studies have verified and identified several clinical characteristics that are significant predictors of NAFLD, including male sex and increased waist circumference, age, hemoglobin A1c (HbA1c), BMI, AST, alkaline phosphatase, high-density lipoprotein (HDL)-cholesterol, triglycerides, and diastolic blood pressure [51,52].

## 4. How to Pursue a Diagnosis

Evidence of steatosis is required for a formal diagnosis of NAFLD across guidelines [31]. There are various noninvasive tests that can be used, each harboring their own advantages and disadvantages, with several key diagnostic tools discussed below.

### 4.1. Conventional US

Conventional US is commonly used and accepted as a first-line diagnostic tool for steatosis [22,53,54]. A US can reliably and accurately diagnose a moderate-to-severe fatty liver [55] and is widely available, relatively inexpensive, noninvasive, and radiation-free [53]. Despite its advantages, however, US has limited sensitivity in patients with low levels of liver fat (<10%) [56,57,58], meaning such patients with NAFLD are often not diagnosed. In addition, obesity can increase the technical difficulty of conducting a US, there is potential for inter/intraobserver variability in US interpretation, and the wait times for imaging can be lengthy in primary care [58,59,60]. For these reasons, PCPs may choose not to use conventional US as a first-line tool (Box 2); nonetheless, conventional US remains an important diagnostic tool for NAFLD diagnosis in primary care.

Box 2 **Do all patients need a liver ultrasound for diagnosing NAFLD?**

   The guidelines recommend that evidence of steatosis is required for diagnosing NAFLD, with conventional US being recommended as a first-line diagnostic tool, as it is cheaper and more widely available than other imaging modalities, specifically, controlled attenuation parameter (CAP) and magnetic resonance imaging (MRI) methods (listed below). When NAFLD is suspected, visualizing steatosis provides PCPs and patients with a confirmatory result, which offers a level of certainty that will prompt a management plan.

   Alternatively, initially recommending lifestyle changes prior to a confirmatory diagnosis allows for earlier intervention and avoids the costs and waiting times associated with diagnostic tests. If a patient shows improvement with lifestyle changes (e.g., a lowered ALT level and, if relevant, lowered triglyceride and/or HbA1c levels, alongside weight loss), a confirmatory test may not be needed, as the parallel improvements in several measures lend strong confidence to the diagnosis of NAFLD. A weight-loss--first approach can be considered when patients have features of metabolic dysfunction. However, more extensive testing should be considered in patients without these risk factors for NAFLD, or when weight loss does not improve ALT, triglyceride, and/or HbA1c levels.

### 4.2. CAP Method

CAP is a measure of liver steatosis that is obtained through the use of a transient elastography device, which is an accurate, noninvasive, and feasible technique, with a value of >275 dB/m having good sensitivity for detecting steatosis [53]. However, the limitations of CAP include its suboptimal performance for quantifying steatosis, in which it is outperformed by MRI-proton density fat fraction [53]; high skin-to-capsule distance potentially, causing an overestimation of steatosis level [61]; and CAP measurements potentially being affected by the intake of meals prior to the examination, meaning that there may be precedent for patients to fast for a minimum of 150 min prior to examination [62]. Furthermore, due to the limited availability of CAP and lack of head-to-head studies with a US, conventional US remains the recommended first-line diagnostic tool for NAFLD [53].

### 4.3. MRI Methods

MRI methods, including magnetic resonance spectroscopy (MRS), provide a means to diagnose steatosis quantitatively. These methods have high diagnostic accuracy, including for low-grade steatosis (5–33%), and have low interobserver variability compared with other imaging modalities [58,63,64,65]. However, a high cost and limited availability mean they are not widely used for diagnosing steatosis in routine clinical care [22]. The guidelines state that MRI and MRS are more suitable in clinical research and trial settings [9,22,53,54].

### 4.4. Noninvasive Scores

Several noninvasive scores composed of clinical and laboratory parameters have been developed for predicting steatosis; examples include the SteatoTest™, the fatty liver index, the hepatic steatosis index, the lipid accumulation product index, and the NAFLD liver fat score (see review [66]). However, these scores are not recommended by the guidelines for diagnosing NAFLD due to their limited accuracy and availability, and, thus, their discussion is beyond the scope of this review [9,22,53,54].

**Case Study.** Assessment history: You receive notes from 2 years ago from the patient’s previous physician (Figure 1), as follows:

- Previous values: Age: 38, BMI: 27.0 kg/m^2^, waist circumference: 38 inches, blood pressure: 136/87 mmHg, and consuming 7 units of alcohol per week;

− Current values: Age: 40, BMI: 32.3 kg/m^2^, waist circumference: 42 inches, blood pressure: 138/86 mmHg, and consuming 7 units of alcohol per week;

- Previous values: Cholesterol: 105 mg/dL, triglyceride: 32 mg/dL, and HDL-cholesterol: 22 mg/dL;

− Current values: Cholesterol: 210 mg/dL, triglyceride: 174 mg/dL, HDL-cholesterol: 31 mg/dL, and LDL-cholesterol: 144 mg/dL;

- Previous values: ALT: 28 U/L and AST: 24 U/L;

− Current values: ALT: 54 U/L, AST: 44 U/L, and platelets: 220 K.

You note that the patient must have undergone substantial weight gain (~5 BMI units) alongside the recent onset of abnormal aminotransferases.

Approach 1

Presuming a diagnosis of NAFLD, you offer options for weight loss and recommend that they reduce their intake of refined sugar and alcohol and try to cut caloric intake in general in order to help aid weight loss.

Approach 2

In line with the current and previous results, you screen the patient for cardiovascular risk and liver disease (Figure 1), with the following results:

- Atherosclerotic cardiovascular disease (ASCVD) risk score: 2.8%, with a 10-year risk of an ASCVD event;
- Negative viral hepatitis B and C testing;
- Liver ultrasound (US) demonstrating hepatic steatosis;
- Fibrosis-4 (Fib-4): 1.09 (low risk).

You diagnose the patient with NAFLD, provide options for weight loss, and recommend that they reduce their intake of sugar and alcohol.

## 5. Interventions: What Are the Options?

Once NAFLD is diagnosed, the care interventions include prescriptions for weight loss, cardiovascular risk management, advanced fibrosis risk assessments, and referral to a hepatologist.

### 5.1. Weight-Loss Interventions

Lifestyle interventions aimed at weight loss are key to the management of NAFLD (Figure 2) and fall under the following two main categories: increased physical activity and diet. Increasing physical activity on its own, including both aerobic and resistance exercise, can reduce liver fat content [67,68]. The benefits of exercise on liver fat content are more pronounced the higher an individual’s BMI [69]. Even in the absence of weight loss or dietary changes, exercise has been shown to reduce liver fat content, markers of liver disease (ALT and AST), and lipid levels [69,70,71,72,73]. Exercise is, therefore, a key recommended intervention in NAFLD [9,21,49,74], with 30–60 min of activity three to four times per week having been shown to improve liver fat content [74]. However, exercise alone seldom leads to sustained weight loss, and many people are not able to sustain large elevations in activity levels [75]. That said, even modest sustained increases in activity levels offer some benefits, as noted above, and may help to prevent weight regain after diet-induced weight loss. It can also be easier for some individuals to be more active following weight loss.

Dietary intervention is an important care intervention in NAFLD [9,21,49,74] and, combined with exercise, may be more effective in reducing liver fat content than exercise alone [70]. Hypocaloric diets and the Mediterranean diet have been shown to improve liver fat content and levels of aminotransferase and inflammatory markers [76,77,78]. As dietary changes are one of the key factors in preventing and reversing NAFLD, this is an area where physicians should support their patients by offering a range of options. Often, trial and error may be needed to find the optimal dietary changes for an individual patient. Discussing the variety of options available to patients is critical to devising a realistic management plan that is tailored to the patient’s needs and preferences, with numerous tools now at hand to help them to lose weight.

### 5.2. Cardiovascular Risk Management

The cardiovascular/metabolic risk factors for NAFLD include T2D, obesity, hypertension, and dyslipidemia [31]. In turn, patients with NAFLD are at an increased risk of cardiovascular events [79], including myocardial infarction, ischemic stroke, atrial fibrillation, heart failure, coronary artery disease, hypertension, and atherosclerosis [80,81], compared to patients without NAFLD. Moreover, patients with NAFLD with T2D, obesity, hypertension, and dyslipidemia are at an increased risk of advanced liver fibrosis, NASH, cirrhosis, and liver-related and overall mortality [13,14,15,16]. Hence, monitoring patients with NAFLD for cardiovascular risk factors and events is important (Figure 2). A tight association has been identified between NAFLD and T2D; as such, several anti-diabetic drugs, such as glucagon-like peptide-1 receptor agonists, thiazolidinedione insulin sensitizers, and sodium/glucose cotransporter-2 inhibitors, have been the subject of clinical trials for NAFLD and have shown potential for improving the outcomes in patients with NAFLD, both with and without comorbid T2D [82]. Various tools are available to physicians to calculate cardiovascular disease risk, such as the Atherosclerotic Cardiovascular Disease Risk Estimator Plus (ASCVD Risk Estimator; https://tools.acc.org/ascvd-risk-estimator-plus/#!/calculate/estimate/ (accessed on 25 March 2022)) and HeartScore (HeartScore^®^; https://www.heartscore.org/en_GB (accessed on 25 March 2022)) (Table 1). For managing patients at risk of cardiovascular disease, the guidelines recommend use of statins, as they do not present any safety issues in patients with NAFLD [9,21]. Finally, weight-loss interventions can also improve the cardiovascular risk profiles of patients with NAFLD [83,84].

**Case Study.** Six-month follow-up: Your patient returns six months later for a follow-up, having lost 8 kg with dietary changes and an alcohol reduction (Figure 1), as follows:

- BMI: 29.7 kg/m^2^, waist circumference: 39.5 inches, blood pressure: 126/78 mmHg, and consuming 3 units of alcohol per week;
- Cholesterol: 190 mg/dL, triglyceride: 158 mg/dL, HDL-cholesterol: 35 mg/dL, and LDL-cholesterol: 123 mg/dL;
- ALT: 32 U/L, AST: 30 U/L, and platelets: 225 K;
- HbA1c: 5.8%;
- Fib-4: 0.94 (low risk);
- ASCVD risk score: 1.6%, with a 10-year risk of an ASCVD event.

You congratulate your patient on their healthy lifestyle changes and encourage them to continue their healthy behaviors.

**Case Study**. Five years later.

Your patient returns after being lost to follow-up for 5 years (now age 45). During that time, they have gained 22 kg since their last visit (Figure 1), with the following results:

- BMI: 37.0 kg/m^2^, waist circumference: 44 inches, blood pressure: 151/88 mmHg, and consuming 10 units of alcohol per week;
- Cholesterol: 225 mg/dL, triglyceride: 220 mg/dL, HDL-cholesterol: 30 mg/dL, and LDL-cholesterol: 151 mg/dL;
- ALT: 64 U/L, AST: 60 U/L, and platelets: 165 K;
- HbA1c: 6.4%;
- Fib-4: 2.05 (indeterminate risk);
- ASCVD risk: 6.2%, with 10-year risk of an ASCVD event.

In this visit, you recognize the changes in the metabolic profile accompanying the increases in weight and alcohol intake. It is important to note the increased risk of advanced fibrosis by Fib-4 and the climbing ASCVD risk, as well as the increased risk of diabetes by the elevated HbA1c levels. You recommend weight loss with a range of evidence-based dietary options and a reduced alcohol intake. You order a confirmatory advanced fibrosis risk assessment with VCTE or consider whether to refer the patient to a hepatologist. You also note the raised blood pressure and make a note to check this again at the next follow-up, having recommended lifestyle changes including reducing their salt intake.

### 5.3. Fibrosis Risk Assessments

Advanced fibrosis is the main prognostic factor for liver-related morbidity and mortality in NAFLD and should, therefore, be assessed in a primary care setting once a diagnosis has been made (Figure 2) [30,34,35,36,37]. A liver biopsy remains as the gold standard for fibrosis assessment [9,22,53,54]; however, it is invasive, expensive, and shows variability in interpretation [9,22,54]. Thus, a liver biopsy is impractical in a primary care setting. Over the past two decades, noninvasive fibrosis risk assessments have emerged that can facilitate fibrosis risk prediction in primary care and identify patients who are in need of a referral to a hepatology specialist.

#### Noninvasive Scores for Fibrosis

*Serologic Tests: Fibrosis-4 (Fib-4) Index and NAFLD Fibrosis Score (NFS)*. The Fib-4 index is a score that is calculated based on a patient’s age, aminotransferase levels (AST and ALT), and platelet count (Fibrosis-4 Score) [85] (Table 1), the latter of which can be obtained by performing a routine blood test [86]. Fib-4, therefore, represents a relatively-easy-to-implement score in a primary care setting (Box 3). It is validated for screening patients at risk of fibrosis and has a high negative predictive value in detecting advanced fibrosis in low-risk populations, meaning that it can accurately exclude advanced fibrosis [86,87,88,89].

NFS is another, slightly more complex, scoring system that is based on routinely collected demographic, clinical, and laboratory variables (such as age, BMI, presence of impaired fasting glycemia/diabetes, AST/ALT ratio, platelet count, and albumin levels) (NAFLD fibrosis score calculator (nafldscore.com (accessed on 1 December 2021)) [90] (Table 1). NFS is validated for diagnosing fibrosis stages and diagnosing/excluding advanced fibrosis in patients with NAFLD [90].

Several guidelines recommend using Fib-4 (Box 4) as the first-line noninvasive scoring system to screen for fibrosis in clinical practice [9,53,74,91,92], due to its low cost, wide availability, and ability to accurately exclude advanced fibrosis [93,94]. Meta-analyses show that Fib-4 and NFS outperform other commonly used noninvasive scores such as the AST/platelet ratio index and the BARD (BMI ≥ 28 = 1 point, AST/ALT ratio of ≥ 0.8 = 2 points, diabetes mellitus = 1 point) score in predicting fibrosis progression, liver-related events, and mortality [95,96]. The limitations of Fib-4 and NFS include low sensitivity, differing predictive probabilities, the risk of false positives, and reduced accuracy with increasing steatosis severity, patient age, obesity, and diabetes [88,97,98,99,100].

Box 3 **When to check Fib-4 score.**

   Assessing for advanced fibrosis is a critical component of NAFLD management once the diagnosis has been made. Fib-4 is a high-performing, cheap, and easily accessible test based on readily available lab parameters and is the cornerstone of the most recent guidelines for NAFLD fibrosis risk assessment in clinical care [24]. The guidelines recommend reassessing Fib-4 scores every 2 to 3 years, including in low-risk patients [24,43]. Checking scores every year may help to identify patients who are at risk of advanced fibrosis/NASH earlier (Box 2); however, this would increase the cost and risk of false positives. Doctors should take a pragmatic approach and assess Fib-4 depending on patients’ progress. When, for example, weight loss is sustained and Fib-4 scores have previously declined, the need to repeat Fib-4 testing would be lower compared to an individual who has gained weight and had elevated Fib-4 scores in the past.

*Serologic Test: Enhanced Liver Fibrosis (ELF™)*. The ELF™ test is a blood test that measures three markers of liver fibrosis (hyaluronic acid, procollagen III amino-terminal peptide, and the tissue inhibitor of matrix metalloproteinase 1), from which a score is computed [101]. The ELF™ test can accurately diagnose fibrosis stages [102,103,104]. In low-risk populations, it has a high negative predictive value but low positive predictive value in detecting advanced fibrosis, meaning that it can accurately exclude, but not diagnose, advanced fibrosis in low-prevalence settings [105]. The National Institute for Health and Care Excellence guidelines recommend using ELF™ to assess advanced liver fibrosis, citing its accuracy and cost-effectiveness [49,54]. However, ELF™ is not recommended by the American Association for the Study of Liver Diseases or the Asia–Pacific Working Party guidelines for clinical use, due to its limited availability [9,22]. The European Association for the Study of Liver (EASL) guidelines recommend its use but acknowledge this limitation [53].

*Imaging: Vibration-controlled Transient Elastography (VCTE)*. VCTE using a controlled attenuation parameter measured with FibroScan^®^ represents a US-based tool for assessing liver stiffness and, in turn, fibrosis. It is relatively quick (~10–20 min) and easy to perform by trained individuals, producing reliable and reproducible results that are immediately available [22,106,107,108]. VCTE is validated for diagnosing fibrosis stages, its diagnostic accuracy increases alongside fibrosis severity, and it can exclude advanced fibrosis [109,110,111]. For detecting the fibrosis stages, VCTE has shown a greater sensitivity than Fib-4 and NFS and a greater specificity than ELF™, but lower accuracy than MRI methods [87,96,102]. However, given the cost and limited availability of MRI methods, these are not recommended for first-line use in clinical practice [31]. VCTE is widely recommended for diagnosing/excluding advanced fibrosis in clinical practice [9,22,53]. According to the EASL Clinical Practice Guidelines, VCTE is recommended for diagnosing/excluding advanced fibrosis in patients with intermediate- and high-risk Fib-4 scores, with the EASL recommending a <8–10 kPa threshold for excluding advanced fibrosis [53]. The limitations of VCTE include uncertainty regarding cutoffs [22] and a reduced diagnostic accuracy in the lower fibrosis stages, in patients with obesity, and with limited operator experience [109,112]. It should also be noted that VCTE is primarily available to specialists, although a recent portable version has been developed for, and is available in, primary care [113].

Box 4 **Fib-4 or NFS?**

   Fib-4 is more cost-effective than NFS for diagnosing advanced fibrosis [54,114]. Meta-analyses show NFS is more accurate in predicting the risk of mortality [115], while Fib-4 is more accurate in diagnosing fibrosis and fibrosis stages [116,117]. One meta-analysis found similar prognostic accuracies for Fib-4 and NFS in terms of liver-related events and mortality, but inconsistent accuracies for predicting the fibrosis stages [95]. Given the cost-effectiveness and diagnostic accuracy of Fib-4 compared with NFS, Fib-4 represents a valuable first-line noninvasive scoring method and has emerged as the preferred initial test for advanced fibrosis in recent guidelines [91,92].

### 5.4. Referral to a Hepatologist

If Fib-4, NFS, ELF™, or VCTE (if performed) suggest that the patient is at high risk of NAFLD with fibrosis (Table S1), the patient should be referred to a hepatologist (Figure 2) [24,43]. Hepatologists can then evaluate the need for additional assessments or a liver biopsy. In patients with noninvasive test results indicating a low risk for advanced fibrosis, the recent guidelines recommend repeating a Fib-4 assessment every 1–2 years in patients with type 2 diabetes, pre-diabetes, or ≥2 metabolic risk factors. These guidelines also recommend that all other patients with NAFLD have a repeat Fib-4 assessment every 2–3 years [91,92]. More evidence is needed to determine the optimal timing of repeat fibrosis assessments.

**Case Study.** Long-term prognosis: Your patient’s (vibration-controlled transient elastography) VCTE results come in, showing that they are at high risk for advanced fibrosis and, in turn, poor future outcomes (Figure 1), with the following result:

- VCTE: 9.9 kPa (high risk).

You refer your patient to a hepatologist and continue to reinforce weight loss and cardiovascular risk reduction as you co-manage this patient.

## 6. Conclusions

PCPs have various tools available at their fingertips for early diagnosis and management of NAFLD (a form of ectopic fat) in primary care settings. In this review, a diagnostic flow chart demonstrates the key points for assessment to identify NAFLD and those at risk. Suspecting and identifying NAFLD is important in developing a management plan, as this enables PCPs to better advocate the need for weight-loss interventions and address cardiovascular risk factors, if not previously carried out. In patients who are at high risk of advanced fibrosis, such as men or those with metabolic risk factors, or in those where NAFLD does not improve through lifestyle change, PCPs should perform advanced fibrosis risk assessments using noninvasive blood-based scores (Fib-4 or NFS) or other evidence-based scores (ELF™ or VCTE) to identify patients with elevated scores who are most in need of a referral to hepatologists for further investigation. Lifestyle modifications early on can improve patient outcomes and may mitigate disease progression, including for both NAFLD and cardiovascular conditions. As such, weight-loss interventions and increased awareness of NAFLD, as well as what risk patterns may constitute NAFLD, are key to identifying and then managing patients with NAFLD in primary care.

## Figures and Tables

**Table 1:** Risk and prediction models.

Model	Risk/Prediction of Disease/Disease Stage	Predictors
ASCVD Risk Estimator	10-year ASCVD risk intended for patients with LDL-cholesterol < 190 mg/dL (4.92 mmol/L), without ASCVD	Current age (years)
		Sex (male/female)
		Race (White/African American/other)
		Systolic blood pressure (mmHg)
		Diastolic blood pressure (mmHg)
		Total cholesterol (mg/dL)
		HDL-cholesterol (mg/dL)
		LDL-cholesterol (mg/dL)
		History of diabetes (yes/no)
		Smoker (current/former/never)
		On hypertension treatment (yes/no)
		On a statin (yes/no)
		On aspirin therapy (yes/no)
HeartScore^®^	10-year risk of first-onset cardiovascular disease in European populations	Risk region (low risk/moderate risk/high risk/very high risk)
		Age (years)
		Sex (male/female)
		Systolic blood pressure (mmHg)
		Total cholesterol (mmol/L or mg/dL)
		HDL-cholesterol (mmol/L)
		Current smoker (yes/no)
Fibrosis-4 score	Prediction of liver fibrosis and cirrhosis	(Age (years) × AST (IU/L))/(Platelet count (10^9^/L) × (square root (ALT (IU/L)))
NAFLD fibrosis score	Prediction of advanced fibrosis	−1.675 + 0.037 × age (years) + 0.094 × BMI (kg/m^2^) + 1.13 × IFG/diabetes (yes = 1, no = 0) + 0.99 × AST/ALT ratio − 0.013 × platelet (×10^9^/L) − 0.66 × albumin (g/dL)

ALT, alanine aminotransferase; ASCVD, atherosclerotic cardiovascular disease; AST, aspartate aminotransferase; BMI, body mass index; HDL, high-density lipoprotein; IFG, impaired fasting glucose; LDL, low-density lipoprotein.

## Data Availability

No new data were created or analyzed in this study. Data sharing is not applicable to this article.

## Supplementary Materials

The following supporting information can be downloaded at: https://www.mdpi.com/article/10.3390/jcm12124001/s1, Table S1: Cutoff values for fibrosis assessments.
