# Supplementary material for: Identifying Patients with Nonalcoholic Fatty Liver Disease in Primary Care: How and for What Benefit?
Source: J Clin Med. 2023 Jun 12;12(12):4001. doi: 10.3390/jcm12124001 (PMC10298910; doi:10.3390/jcm12124001)
Supplement: Supplementary file 1 [file jcm-12-04001-s001.zip › jcm-2284046-supplementary.pdf]

Journal of
Clinical Medicine

MDPI

Supplementary Materials

Table S1. Cutoff values for fibrosis assessments.

	Low Risk for Advanced Fibrosis	Indeterminate Risk for Advanced Fibrosis	High Risk for Advanced Fibrosis	Reference
Fib-4	<1.3	1.3–2.67	>2.67	Kanwal, F.; Shubrook, J.H.; Adams, L.A.; et al. Clinical care pathway for the risk stratification and management of patients with nonalcoholic fatty liver disease. *Gastroenterology* 2021, *161*, 1657–1669 [1].
	≤1.3	1.3–3.25	>3.25	Newsome, P.N.; Cramb, R.; Davison, S.M.; et al. Guidelines on the management of abnormal liver blood tests. *Gut* 2018, *67*, 6–19 [2].
	≤2.67	≤2.67	>2.67	National Institute for Health and Care Excellence. How should I assess a person with NAFLD? Available online (accessible only in the UK): https://cks.nice.org.uk/topics/non-alcoholic-fatty-liver-disease-nafld/diagnosis/assessment/#:~:text=Fibrosis%20%28FIB%29-4%20Score%20%28FIB-4%29%20%E2%80%94%20a%20score%20of,a%20hepatology%20specialist%20for%20further%20assessment%20and%20management (accessed 3 April 2023) [3].
	<1.6	1.6–3.6	>3.6	Kim, B.K.; Kim, D.Y.; Park, J.Y.; et al. Validation of FIB-4 and comparison with other simple noninvasive indices for predicting liver fibrosis and cirrhosis in hepatitis B virus-infected patients. *Liver Int.* 2010, *30*, 546–553 [4].
NFS	≤−1.455	−1.455–0.675	>0.675	Newsome, P.N.; Cramb, R.; Davison, S.M.; et al. Guidelines on the management of abnormal liver blood tests. *Gut* 2018, *67*, 6–19 [2].
	<−1.455	−1.455–0.676	>0.676	Angulo, P.; Hui, J.M.; Marchesini, G.; et al. The NAFLD fibrosis score: a noninvasive system that identifies liver fibrosis in patients with NAFLD. *Hepatology* 2007, *45*, 846–854 [5].
	≤−1.455	>−1.455	>−1.455	National Institute for Health and Care Excellence. How should I assess a person with NAFLD? Available online: https://cks.nice.org.uk/topics/non-alcoholic-fatty-liver-disease-nafld/diagnosis/assessment/#:~:text=Fibrosis%20%28FIB%29-4%20Score%20%28FIB-4%29%20%E2%80%94%20a%20score%20of,a%20hepatology%20specialist%20for%20further%20assessment%20and%20management (accessed 3 April 2023) [3].
ELF™	≤9.5		>9.5	Newsome, P.N.; Cramb, R.; Davison, S.M.; et al. Guidelines on the management of abnormal liver blood tests. *Gut* 2018, *67*, 6–19 [2].
	<10.5	<10.5	≥10.51	National Institute for Health and Care Excellence. How should I assess a person with NAFLD? Available online: https://cks.nice.org.uk/topics/non-alcoholic-fatty-liver-disease-nafld/diagnosis/assessment/#:~:text=Fibrosis%20%28FIB%29-4%20Score%20%28FIB-4%29%20%E2%80%94%20a%20score%20of,a%20hepatology%20specialist%20for%20further%20assessment%20and%20management (accessed 3 April 2023) [3].
	<7.7	≥7.7–<9.8	≥9.8	Siemens Healthineers. The enhanced liver fibrosis (ELF) blood test: literature compendium volume 1. Available online: https://www.siemens-healthineers.com/en-uk/laboratory-diagnostics/assays-by-diseases-conditions/liver-disease/elf-test (accessed 3 April 2023) [6].
VCTE	<8 kPa	8–12 kPa	>12 kPa	Kanwal, F.; Shubrook, J.H.; Adams, L.A.; et al. Clinical care pathway for the risk stratification and management of patients with nonalcoholic fatty liver disease. *Gastroenterology* 2021, *161*, 1657–1669 [1].
	≤8.2 kPa[a]		>8.2 kPa[a]	Eddowes, P.J.; Sasso, M.; Allison, M.; et al. Accuracy of FibroScan controlled attenuation parameter and liver stiffness measurement in assessing steatosis and fibrosis in patients with nonalcoholic fatty liver disease. *Gastroenterology* 2019, *156*, 1717–1730 [7].

≤7.8 kPa	>7.8 kPa or invalid scan	Newsome, P.N.; Cramb, R.; Davison, S.M.; et al. Guidelines on the management of abnormal liver blood tests. *Gut* 2018, *67*, 6–19 [2].

[a] Youden's index cutoff value of >8.2 kPa is the threshold for ≥fibrosis stage 2, representing clinically significant fibrosis. ELF™, enhanced liver fibrosis; Fib-4, Fibrosis-4; NFS, NAFLD fibrosis score; VCTE, vibration controlled transient elastography.

References

1. Kanwal, F.; Shubrook, J.H.; Adams, L.A.; Pfotenhauer, K.; Wong, V.W.; Wright, E.; Abdelmalek, M.F.; Harrison, S.A.; Loomba, R.; Mantzoros, C.S.; et al. Clinical care pathway for the risk stratification and management of patients with nonalcoholic fatty liver disease. *Gastroenterology* **2021**, *161*, 1657–1669. https://doi.org/10.1053/j.gastro.2021.07.049.
2. Newsome, P.N.; Cramb, R.; Davison, S.M.; Dillon, J.F.; Foulerton, M.; Godfrey, E.M.; Hall, R.; Harrower, U.; Hudson, M.; Langford, A.; et al. Guidelines on the management of abnormal liver blood tests. *Gut* **2018**, *67*, 6–19. https://doi.org/10.1136/gutjnl-2017-314924.
3. National Institute for Health and Care Excellence. How should I assess a person with NAFLD? Available online (accessible only in the UK): https://cks.nice.org.uk/topics/non-alcoholic-fatty-liver-disease-nafld/diagnosis/assessment/#:~:text=Fibrosis%20%28FIB%29-4%20Score%20%28FIB-4%29%20%E2%80%94%20a%20score%20of,a%20hepatology%20specialist%20for%20further%20assessment%20and%20management (accessed 3 April 2023).
4. Kim, B.K.; Kim, D.Y.; Park, J.Y.; Ahn, S.H.; Chon, C.Y.; Kim, J.K.; Paik, Y.H.; Lee, K.S.; Park, Y.N.; Han, K.H. Validation of FIB-4 and comparison with other simple noninvasive indices for predicting liver fibrosis and cirrhosis in hepatitis B virus-infected patients. *Liver Int* **2010**, *30*, 546–553. https://doi.org/10.1111/j.1478-3231.2009.02192.x.
5. Angulo, P.; Hui, J.M.; Marchesini, G.; Bugianesi, E.; George, J.; Farrell, G.C.; Enders, F.; Saksena, S.; Burt, A.D.; Bida, J.P.; et al. The NAFLD fibrosis score: A noninvasive system that identifies liver fibrosis in patients with NAFLD. *Hepatology* **2007**, *45*, 846–854. https://doi.org/10.1002/hep.21496.
6. Siemens Healthineers. The enhanced liver fibrosis (ELF) blood test: Literature compendium volume 1. Available online: https://www.siemens-healthineers.com/en-uk/laboratory-diagnostics/assays-by-diseases-conditions/liver-disease/elf-test (accessed 3 April 2023).
7. Eddowes, P.J.; Sasso, M.; Allison, M.; Tsochatzis, E.; Anstee, Q.M.; Sheridan, D.; Guha, I.N.; Cobbold, J.F.; Deeks, J.J.; Paradis, V.; et al. Accuracy of FibroScan controlled attenuation parameter and liver stiffness measurement in assessing steatosis and fibrosis in patients with nonalcoholic fatty liver disease. *Gastroenterology* **2019**, *156*, 1717–1730. https://doi.org/10.1053/j.gastro.2019.01.042.
